# The molecular motor Myosin Va interacts with the cilia-centrosomal protein RPGRIP1L

**DOI:** 10.1038/srep43692

**Published:** 2017-03-07

**Authors:** L. H. P. Assis, R. M. P. Silva-Junior, L. G. Dolce, M. R. Alborghetti, R. V. Honorato, A. F. Z. Nascimento, T. D. Melo-Hanchuk, D. M. Trindade, C. C. C. Tonoli, C. T. Santos, P. S. L. Oliveira, R. E. Larson, J. Kobarg, E. M. Espreafico, P. O. Giuseppe, M. T. Murakami

**Affiliations:** 1 Brazilian Biosciences National Laboratory, National Center for Research in Energy and Materials, Campinas, SP, Brazil; 2 Graduate Program in Functional and Molecular Biology, Institute of Biology, University of Campinas, Campinas, SP, Brazil; 3 Department of Cell and Molecular Biology, Faculty of Medicine of Ribeirão Preto, University of São Paulo, Ribeirão Preto, SP, Brazil; 4 Department of Biochemistry and Tissue Biology, Institute of Biology, University of Campinas, Campinas, SP, Brazil

## Abstract

Myosin Va (MyoVa) is an actin-based molecular motor abundantly found at the centrosome. However, the role of MyoVa at this organelle has been elusive due to the lack of evidence on interacting partners or functional data. Herein, we combined yeast two-hybrid screen, biochemical studies and cellular assays to demonstrate that MyoVa interacts with RPGRIP1L, a cilia-centrosomal protein that controls ciliary signaling and positioning. MyoVa binds to the C2 domains of RPGRIP1L via residues located near or in the Rab11a-binding site, a conserved site in the globular tail domain (GTD) from class V myosins. According to proximity ligation assays, MyoVa and RPGRIP1L can interact near the cilium base in ciliated RPE cells. Furthermore, we showed that RPE cells expressing dominant-negative constructs of MyoVa are mostly unciliated, providing the first experimental evidence about a possible link between this molecular motor and cilia-related processes.

Class V myosins are motor proteins that transport and/or tether vesicles, organelles and macromolecules, using the energy of ATP hydrolysis to walk toward the plus end of actin filaments^1^. They are found from fungi to vertebrates^2^ and are involved in important cellular processes such as organelle inheritance in budding yeast^3^ and organelle transport into neuronal dendritic spines^4^. Three class V myosin genes (*MYO5A, MYO5B*, and *MYO5C*) are present in vertebrates^5^, of which *MYO5A* has crucial roles in melanocytes and neurons^6^.

Loss-of-function mutations in *MYO5A* are associated with the Griscelli syndrome type 1 in humans, characterized by partial albinism and severe neurological disorders^7^. The partial albinism is due to a defect in the capture and transport of melanosomes by the protein myosin Va (MyoVa) in melanocytes^3^,^8^, whereas the neurological impairment has probably pleiotropic origins, considering the several functions reported for MyoVa in the brain^6^. These functions include regulation of the exocytosis of large dense-core vesicles^9^,^10^, the transport of endoplasmic reticulum into Purkinje cell dendritic spines^11^ and the targeting of proteins involved in signaling pathways that control neuronal cell size and shape, such as PTEN^12^ and RILPL2^13^.

Interestingly, PTEN and RILPL2 have been demonstrated to control cilia assembly/disassembly and to regulate ciliary membrane content, respectively^14^,^15^. Cilia are microtubule-based organelles that emerge from the centrosome to form a cell surface projection when cells exit mitosis^16^. Neuronal cells usually exhibit a single non-motile cilium, called primary cilium, which modulates key processes such as neurogenesis, cell polarity, axonal guidance and possibly adult neuronal function^17^. Besides PTEN and RILPL2, other binding partners of MyoVa, such as the small GTPases Rab11 and Rab8, also play a role in cilia, by coordinating the assembly of the primary cilium membrane^18^. However, whether MyoVa participates in processes related to the primary cilium function has not previously been investigated.

Studies on several cell lines have shown that a subpopulation of MyoVa localizes to the centrosome during interphase and to the mitotic spindle poles and fibers during cell division^19^,^20^,^21^,^22^. These have been intriguing observations, because, for several decades, the centrosome was viewed as a center devoted to nucleate, anchor and release microtubules^23^. However, this paradox has been changing with the recent discovery that the centrosome is also an actin-organizing center^24^, which correlates with the abundant presence of the actin-based motor MyoVa at this organelle.

The centrosomal targeting of MyoVa depends on its globular tail domain (GTD)^19^,^20^, but the molecular mechanisms linking this motor protein to the centrosome have been elusive. Here, we show that the GTD of MyoVa binds to the C2 domains of RPGRIP1L, a cilia-centrosomal protein that regulates basal body positioning and ciliary signaling pathways, such as Wnt and sonic hedgehog^25^,^26^,^27^,^28^. Moreover, we provide the first evidence that dominant-negative constructs of MyoVa interfere with ciliogenesis, paving new connections between the actin-based transport machinery and centrosome-regulated processes.

## Results

### MyoVa-GTD interacts with the C2 domains of RPGRIP1L

**Figure 1:**
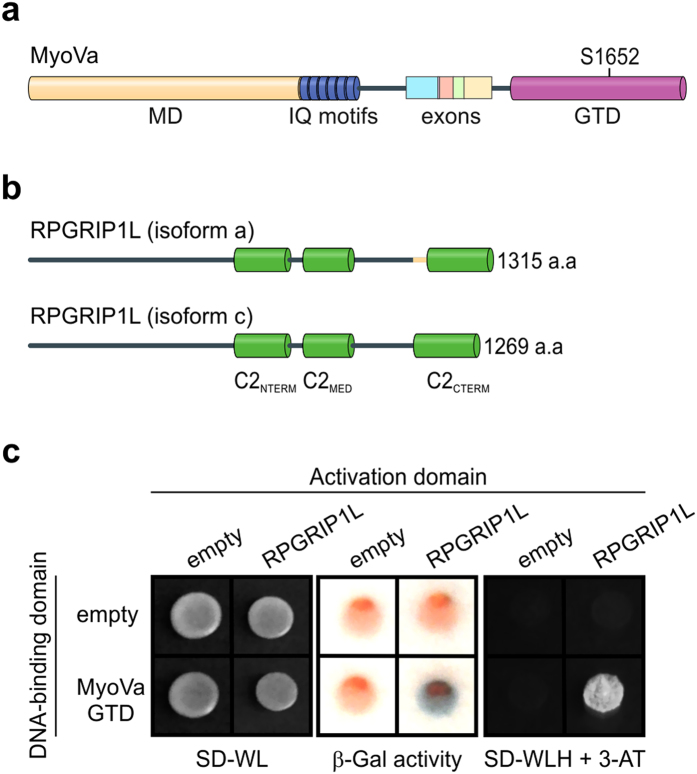
MyoVa interacts with the isoform c of RPGRIP1L. (**a**) Schematic representation of the human MyoVa domain architecture highlighting the Motor domain (MD, *yellow*), the IQ motifs (*blue*), the alternatively spliced exons at the medial tail (A–E, exon F was omitted), and the GTD (*pink*) as well as the phosphorylation site at S1652 (top). (**b**) Comparison between the longest isoform of RPGRIP1L (isoform a) with that identified in this work (full-length isoform c). The isoform a is encoded by all the 27 exons of *RPGRIP1L* gene whereas isoform c lacks 46 residues (*yellow*) encoded by the exon 23. Both isoforms contain three C2 domains (*green*). (**c**) Activation of the *LacZ* and *HIS3* reporter genes in YTH assays indicates that MyoVa-GTD interacts with the isoform c of RPGRIP1L. Yeast cells expressing only Gal4 Activation domain and/or LexA DNA-binding domain were used as negative controls.

Yeast two-hybrid (YTH) screen using MyoVa-GTD as bait and a cDNA library of human fetal brain as prey revealed RPGRIP1L, among other proteins, as a potential binding partner of MyoVa (Fig. 1, Supplementary Table S1). The transcript identified in the screening comprises the whole open reading frame of *RPGRIP1L* variant 3 (NCBI accession number: NM_001308334.2). This variant encodes the RPGRIP1L isoform c, a multi-domain protein composed of a region predicted to form coiled-coils followed by three C2 domains, named here as C2_NTERM_, C2_MED_ and C2_CTERM_ (Fig. 1b). Compared to the longest isoform reported for RPGRIP1L (isoform a, NCBI accession number: NP_056087.2), the isoform c lacks only 46 amino-acid residues (encoded by the exon 23) between the last two C2 domains (Fig. 1b).

To validate this interaction in the YTH system, we co-transformed the yeast strain L40 with the bait (pBTM116 or pBTM116_MyoVa-GTD) and prey plasmids (pACT2 or pACT2_RPGRIP1L) and evaluated the activation of two reporter genes, *LacZ* and *HIS3*. As expected, only the colonies expressing both MyoVa-GTD and RPGRIP1L displayed β-galactosidase activity and grew in presence of 10 mM 3-AT, indicating that RPGRIP1L binds to MyoVa-GTD (Fig. 1c).

**Figure 2:**
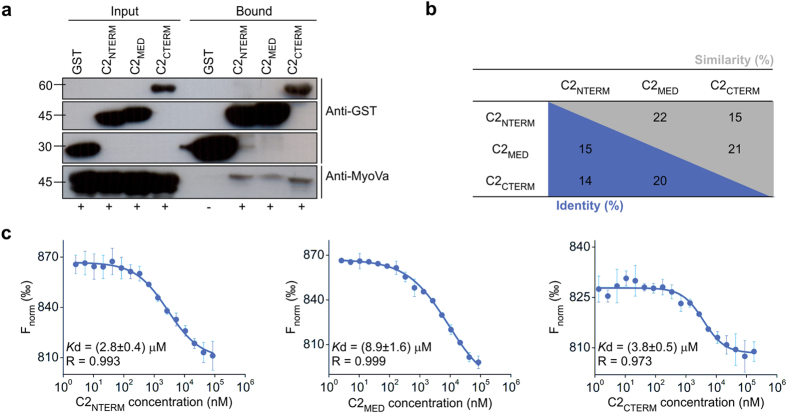
MyoVa-GTD binds to the C2 domains of RPGRIP1L. (**a**) Pull-down assays showing that 6xHis-MyoVa-GTD construct interacts with the three C2 domains of RPGRIP1L fused to GST. Bacteria expressing only 6xHis-MyoVa-GTD and GST were used as negative control. (**b**) Amino acid sequence identity and similarity between the C2 domains of human RPGRIP1L, according to structural alignment of the homology models of C2_MED_ and C2_CTERM_ (predicted using the HHPred server^54^) and the RMN structure of C2_NTERM_ (PDB ID: 2YRB) using the service PDBeFold^55^. (**c**) MST assays showing that MyoVa-GTD binds to C2_NTERM_ (left), C2_MED_ (center) and C2_CTERM_ (right).

As aforementioned, RPGRIP1L contains three C2 domains and there is increasing evidence that they can mediate protein-protein interactions, especially in the ciliary transition zone^28^,^29^. Therefore, to characterize the RPGRIP1L∙MyoVa interaction and to evaluate the role of these C2 domains in MyoVa binding, we performed pull-down assays (Fig. 2a). The three C2 domains were able to interact with MyoVa-GTD, despite their low sequence identity (Fig. 2a,b). Microscale thermophoresis (MST) experiments showed that RPGRIP1L-C2 domains bind to MyoVa-GTD with dissociation constants in the 3–9 μM range, with the C2_NTERM_ and C2_CTERM_ displaying the highest affinity for MyoVa-GTD (Fig. 2c).

### RPGRIP1L binds to a conserved site of MyoVa and Vb GTDs

**Figure 3:**
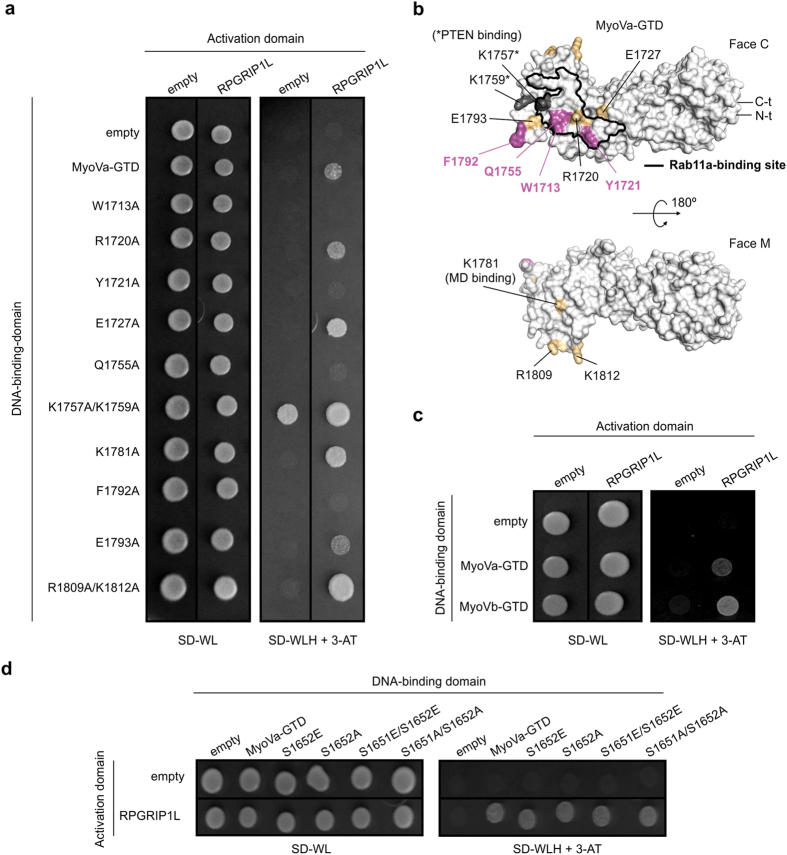
RPGRIP1L binds to a conserved binding site of class V myosins. (**a**) YTH assays showing that mutations on the face C of MyoVa-GTD (W1713A, Y1721A, Q1755A and F1792A) disrupt the interaction between MyoVa-GTD and RPGRIP1L. (**b**) Surface representation of MyoVa-GTD^30^ (PDB ID: 4J5L) highlighting the residues identified as being involved in RPGRIP1L binding (*pink*), and other residues whose Ala mutants displayed auto-activation (K1757A, K1759A; *grey*) or a result similar to the wild-type MyoVa-GTD (*yellow)* in the YTH assay (*panel A*). The Rab11a-binding site, inferred from the crystal structure of MyoVb∙Rab11a complex^33^ (PDB ID: 4LX0), as well as the N- and C-termini of MyoVa-GTD (N-t and C-t) are indicated. (**c**) YTH assays showing that the isoform c of RPGRIP1L also interacts with MyoVb-GTD. Yeast cells expressing only Gal4 Activation domain and/or LexA DNA-binding domain were used as negative controls. (**d**) Phospho-mimetic (Ser to Glu) and non-phospho-mimetic (Ser to Ala) mutants of MyoVa-GTD interacted with RPGRIP1L in YTH assays, similarly to the wild-type protein. Yeast cells expressing only Gal4 Activation domain and/or LexA DNA-binding domain were used as negative controls.

The GTDs of MyoVa and Vb share a protein-binding site at the face C of lobule II, which is also conserved in the class V myosin Myo2p from yeast^30^. To investigate if RPGRIP1L also binds to this region, we mutated some conserved residues at this site to alanine and performed YTH assays (Fig. 3a), following a strategy similar to that used to map the protein-binding sites of Myo2p^31^. Additionally, we evaluated alanine mutants of residues involved in PTEN recognition (K1757 and K1759)^12^. As a control, residues from the other face of the MyoVa-GTD (face M), including one that is crucial for the binding of MyoVa motor domain (K1781) in the auto-inhibited state^32^, were also mutated. Analysis of these mutants indicated that the residues W1713, Y1721, Q1755 and F1792 are required for the interaction between MyoVa and RPGRIP1L (Fig. 3a). Based on these data, we suggest that the RPGRIP1L-binding site overlaps with those of Kar9 and Inp2 to Myo2p and that of Rab11a to MyoVb^31^,^33^ (Fig. 3b). In agreement with this result, YTH assays showed that RPGRIP1L also interacts with MyoVb-GTD (Fig. 3c), indicating a redundant role for MyoVa and Vb in RPGRIP1L binding.

As MyoVa-GTD can be phosphorylated on residue S1652 by calcium/calmodulin-dependent protein kinase II (CaMKII)^34^,^35^, which results in its release from melanosomes and inhibition of melanosome transport^36^, we also investigated whether S1652 phosphorylation could affect RPGRIP1L binding. For this purpose, we used phospho-mimetic (S1652E and S1651E/S1652E) and non-phospho-mimetic (S1652A and S1651A/S1652A) mutations previously validated by Karcher and co-workers^36^. YTH analyses showed that RPGRIP1L was capable to interact with both mimetic mutants, indicating that S1652 phosphorylation does not prevent the binding of RPGRIP1L to MyoVa-GTD (Fig. 3d).

### MyoVa interacts with RPGRIP1L at the centrosome

**Figure 4:**
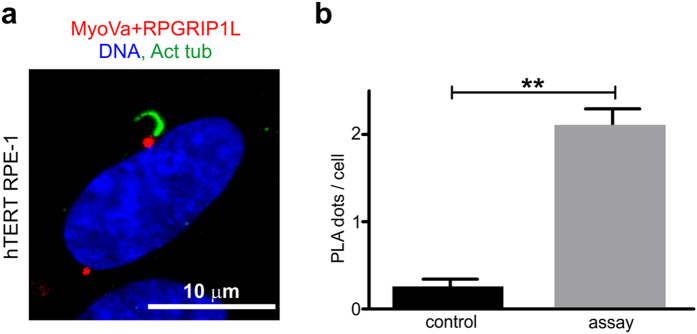
MyoVa interacts with RPGRIP1L at the centrosome. (**a**) PLA indicate the presence of endogenous complexes between MyoVa and RPGRIP1L (*red dots*) in a radius of 2 μm from the center of primary cilium base in 16% of hTERT RPE-1 ciliated cells (n = 522). Primary cilium axoneme is marked with acetylated-α-tubulin antibody (*green*). (**b**) The mean PLA dot count per cell was 2.12 ± 0.17 (mean ± SEM) in the assay and 0.26 ± 0.08 (mean ± SEM) in the control without primary antibodies (*P* < 0.005, two-tailed Student’s *t-*test).

To validate the interaction between endogenous MyoVa and RPGRIP1L, we performed proximity ligation assays (PLA) in RPE cells, a model system for studying primary cilium formation and function^18^. Since it is well known that a pool of MyoVa^19^,^20^,^21^,^22^ and of RPGRIP1L^25^,^28^,^37^ localize at the centrosome, we investigated whether they interact at this microenvironment in ciliated cells. As expected, the presence of PLA dots evidenced the physical interaction between MyoVa and RPGRIP1L near the primary cilium base (Fig. 4), indicating that the binding of MyoVa to RPGRIP1L can occur at the centrosome and might be involved in cilia-related processes.

### Dominant-negative expression of MyoVa inhibits ciliogenesis

**Figure 5:**
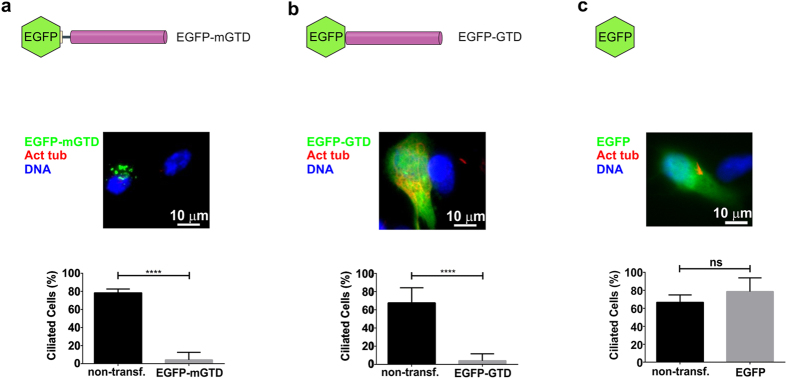
Overexpression of dominant-negative constructs of MyoVa suppressed ciliogenesis. Overexpression of EGFP-mGTD (**a**) or EGFP-GTD (**b**) in transfected RPE cells (n = 31 or 83) severely decreased the proportion of ciliated cells compared to their non-transfected neighbors (n = 170 or 373). Means ± SD represent the average data of 4 (**a**) or 17 (**b**) analyzed fields. (**c**) Overexpression of EGFP alone (negative control) did not affect the proportion of ciliated cells (n = 37) compared to the non-transfected condition (n = 238). Means ± SD represent the average data of eight analyzed fields. ns = nonsignificant; ****P ≤ 0.0001 (two-tailed Student’s *t*-test).

The fact that RPGRIP1L, as well as other MyoVa-binding proteins (PTEN, Rab8, Rab11 and RILPL2), is involved with the regulation of the primary cilium structure and or composition^14^,^15^,^18^,^38^ prompted us to investigate the effect of overexpressing two dominant-negative constructs of MyoVa in ciliogenesis, EGFP-GTD and EGFP-mGTD (GTD + 45 upstream amino-acid residues from the medial tail) (Supplementary Figure S1). Interestingly, these two constructs displayed different distribution patterns, being EGFP-mGTD localized in discrete foci near the nucleus, whereas EGFP-GTD was diffusely distributed at the cytoplasm (Fig. 5a and b), indicating that these additional residues might be critical for GTD targeting. Despite this observation, the overexpression of both constructs strongly suppressed the assembly of primary cilium in RPE cells (Fig. 5a and b), which did not occur in cells expressing EGFP alone (Fig. 5c). The same phenotype was also observed in melanoma B16 cells (data not shown), indicating that MyoVa might play a role in cilia-related processes.

## Discussion

In the present work, we revealed that MyoVa interacts with the cilia-centrosomal protein RPGRIP1L at the centrosome. Moreover, we showed structural features of MyoVa required for RPGRIP1L recognition and provided the earliest evidence for a role of MyoVa in the primary cilium development, a centrosome-regulated process.

We demonstrated *in vitro* that the three C2 domains of RPGRIP1L can recruit the GTD of MyoVa with affinity typical of transient protein-protein interactions (low micromolar *K*_d_), being the C2_NTERM_ and C2_CTERM_ domains those that best recognize MyoVa-GTD. *In vivo*, the affinity between RPGRIP1L and MyoVa might be further enhanced by MyoVa dimerization and the tandem disposition of the three C2 domains in RPGRIP1L, which likely increase the probability of their binding. Attempts to identify residues of RPGRIP1L involved in MyoVa recognition using *in silico* predictions and site-directed mutagenesis were inconclusive, indicating the need of a deeper investigation to elucidate the molecular basis of MyoVa recruitment by the C2 domains of RPGRIP1L (Supplementary Figure S2).

C2 domains are recurrent in ciliary proteins from the transition zone, such as RPGRIP1L^29^ and CC2D2A^39^, as well as in MyoVa-interacting proteins, such as PTEN and the Rab effectors granuphilin-a/b (Gran-a/b) and rabphilin-3A (Rph-3A)^12^,^40^. Although the role of PTEN-C2 domain in MyoVa binding is still elusive^12^, the C2 domains of Gran-a/b and Rph-3A interact with an alternatively spliced region of MyoVa tail, but in a region different from the binding site of RPGRIP1L-C2 domains^40^. Together, these examples illustrate an emerging role for C2 domains in linking proteins to the actin cytoskeleton via the recruitment of MyoVa.

Using YTH assays and site-directed mutagenesis, we showed that RPGRIP1L binds to both MyoVa and Vb, being recognized by a conserved region that overlaps with the Rab11a-binding site^33^. Interestingly, active Rab11 (GTP-bound form) is also recruited to the centrosome – specifically to the mother centriole appendages – where it is “turned off” (GDP-bound form) by Evi5^41^. Inactivation of Rab11a induces the release of effector proteins like MyoVa/Vb, suggesting that, in this microenvironment, the association of MyoVa with RPGRIP1L might be favoured over that with Rab11a. In agreement with this hypothesis, the Rab11a∙GDP binding to MyoVb-GTD monomer^33^ displays a *K*_d_ 6 times higher than that of RPGRIP1L-C2_NTERM_ domain to MyoVa-GTD.

Our results also evidenced a direct interaction between MyoVa and RPGRIP1L at the vicinity of the basal body, indicating that the MyoVa∙RPGRIP1L complex might play a role in primary cilium development. Genetic diseases related to defects in *MYO5A* and *RPGRIP1L* genes are characterized by common neurologic impairments, suggesting they function in correlated pathways in the brain^7^,^25^,^28^,^42^,^43^. One of such pathways might involve the primary cilium, since the protein RPGRIP1L has been linked to signaling pathways that depend on this organelle and play a key role in brain development (sonic hedgehog and Wnt) or brain function (leptin receptor signaling)^26^,^27^,^44^,^45^,^46^. Furthermore, in neuron photoreceptors, RPGRIP1L localizes not only in the connecting cilium but also near the plasma membrane of the calycal processes^28^ – microvillus-like projections rich in actin filaments – suggesting a potential role for RPGRIP1L in anchoring membranes to the actin cytoskeleton via MyoVa.

By overexpressing two dominant-negative constructs of MyoVa in RPE cells, we showed that loss of myosin V transport function suppresses ciliogenesis. Together with the fact that most proteins known to bind to MyoVa GTD regulate cilia assembly (Rab11), transition zone establishment (RPGRIP1L), cilia dynamics (PTEN) or cilia composition (RILPL2), our data support a role for MyoVa in cilia-related processes.

In summary, our studies revealed RPGRIP1L as a novel MyoVa-binding protein – the first to be demonstrated to interact with MyoVa at the centrosome – and uncover an unprecedented link between MyoVa and ciliogenesis, providing new perspectives for studies aiming to better understand why defects in MyoVa cause neurological disorders in Griscelli syndrome patients.

## Methods

### Molecular cloning and site-directed mutagenesis

MyoVa-GTD (residues 1448–1855; NP_000250.3) constructs (wild-type and mutants S1652E, S1652A, S1651E/S1652E, S1651A/S1652A) previously cloned into pET28a tobacco etch virus (TEV) vector^30^ were subcloned into pBTM116 vector between *Eco*RI and *Sal*I restriction sites. The gene region encoding for MyoVb-GTD (residues 1453–1848; NP_001073936.1) was amplified by PCR from a human fetal brain cDNA library (Clontech, Mountain View, CA) and cloned into pBTM116 vector between the *Bam*HI and *Sal*I restriction sites. RPGRIP1L constructs encoding for C2_NTERM_ (residues 561–737), C2_MED_ (residues 781–930) and C2_CTERM_ (residues 1037–1269) were amplified by PCR using as template the pACT2_RPGRIP1L plasmid (NM_001308334.2) identified in the YTH screen described below. RPGRIP1L constructs were cloned into pGEX-4T-1 vector between *Eco*RI and *Not*I restriction sites. MyoVa (W1713A, R1720A, Y1721A, E1727A, Q1755A, K1757A/K1759A, K1781A, F1792A, E1793A, R1809A/K1812A) mutants were generated using the QuikChange site-directed mutagenesis kit (Agilent Technologies, Santa Clara, CA).

### Yeast two-hybrid screen (YTH)

YTH screen was performed in *Saccharomyces cerevisiae* strain L40 (trp1-901, his3D200, leu2–3, ade2 LYS2::(lexAop)4-HIS3 URA3::(lexAop)8-lac GAL4) using MyoVa-GTD cloned into pBTM116 (LexA DNA-binding domain, DBD) as bait and a human fetal brain cDNA library (Clontech) cloned into pACT2 (Gal4 activation domain, AD) as prey. Yeast cells were transformed with pBTM116_MyoVa-GTD vector and the library as described by Alborghetti and co-workers^47^. The screen was performed in solid Synthetic Defined Medium without tryptophan, leucine and histidine (SD-WLH) containing 5 mM 3-amino-1,2,4-triazole (3-AT) (Sigma-Aldrich, St. Louis, MO). To identify the preys, the pACT2 plasmids of positive clones were isolated and sequenced. The DNA sequences were then compared with those available in the NCBI data bank using the BLASTX program^48^. The clone identified as encoding for the full-length RPGRIP1L isoform c (NP_001295263.1) was further selected for *in vitro* and *in cell* validation and characterization.

### Yeast reporter gene assays

To confirm the interaction between pBTM116_MyoVa-GTD and pACT2_RPGRIP1L, *S. cerevisiae* L40 cells were transformed with both constructs. As negative controls, we used L40 cells transformed with pBTM116_MyoVa-GTD and empty pACT2 or pACT2_RPGRIP1L and empty pBTM116. Cells were plated in Synthetic Defined Medium without tryptophan and leucine (SD-WL) and then incubated at 30 °C for 3 days. For β-galactosidase activity assay, cells were transferred to Whatman^®^ 3 MM paper (Sigma-Aldrich), permeabilized with liquid nitrogen and wrapped on a second paper soaked in Z buffer (60 mM Na_2_HPO_4_, 40 mM NaH_2_PO_4_, 10 mM MgCl_2_, 50 mM β-mercaptoethanol and pH 7.0) containing 2 μg/mL 5-bromo-4-chloro-3-indolyl-β-D-galactoside (X-Gal; Sigma-Aldrich). Cells were incubated at 37 °C for a couple of hours until the blue color appears, indicating the β-galactosidase activity. For *HIS3* activation assay, cells were plated in SD-WLH containing 10 mM 3-AT, incubated for 3 days at 30 °C and imaged.

### GST pull-down assays

*Escherichia coli* BL21(DE3)∆SlyD strain cells (containing the pRARE2 plasmid) were transformed with recombinant pET28a-TEV and pGEX-4T-1 to co-express 6xHis-tagged MyoVa-GTD and GST-tagged RPGRIP1L constructs. Cells were cultivated in Terrific Broth (TB) medium containing 100 μg/mL ampicillin, 50 μg/mL kanamycin and 34 μg/mL chloramphenicol. Recombinant protein expression was induced with 0.1 mM isopropyl β-D-1-thiogalactopyranoside (IPTG) (Thermo Fisher Scientific, Waltham, MA) at OD_600_ ~0.6, during 16 h at 18 °C, 200 rpm. Cells were harvested, incubated for 1 h at 4 °C with lysis buffer (50 mM Tris pH 8.0, 250 mM NaCl, 5% *(v/v)* glycerol, 0.01% *(v/v)* tween-20, 1 mM tris(2-carboxyethyl)phosphine (TCEP)) (Sigma-Aldrich) containing 0.1 mg/mL lysozyme (Sigma-Aldrich) and free-EDTA SigmaFast Protease Inhibitor cocktail (Sigma-Aldrich) and lysed in VCX750 Sonics ultrasound (Sonics & Materials, Newtown, CT). The soluble fraction was incubated with Glutathione Sepharose 4B resin (GE Healthcare, Uppsala, Sweden) for 2 h at 4 °C under gentle agitation. Unbound proteins were discarded and resin was washed three times with lysis buffer. Immobilized proteins were eluted with 10 mM reduced glutathione (Sigma-Aldrich) in lysis buffer.

### Immunodetection by Western blot

Proteins were analyzed in 10% SDS-PAGE and transferred to a PVDF Hybond^TM^-P membrane (GE Healthcare) using the Semi-Dry Blotting system (Biorad, Hercules, CA). Membranes were incubated overnight at 4 °C with the following primary antibodies: rabbit anti-MyoVa at 0.4 μg/mL (M4812, Sigma-Aldrich) or mouse anti-GST hybridome (*in house*, Campinas, Brazil)^49^. After overnight incubation, membranes were washed with TBS buffer (50 mM Tris, 150 mM NaCl and pH 7.6) and incubated with the following secondary antibodies diluted in 0.01% *(w/w)* powdered milk in TBS buffer: peroxidase labeled goat anti-rabbit at 0.02 μg/mL (04-15-06, KPL, Gaithersburg, MD) or peroxidase labeled goat anti-mouse at 0.2 μg/mL (401253, Calbiochem, Darmstadt, Germany). Membranes were washed with TBS buffer and incubated with ImmunoCruz^TM^ Luminol reagent (Santa Cruz Biotechnology, Santa Cruz, CA). Immunostaining were visualized using High Performance Chemiluminescence film (GE Heathcare), Carestream^®^ Kodak^®^ autoradiography GBX developer (Carestream, Rochester, NY) and Carestream^®^ Kodak^®^ autoradiography GBX fixer (Carestream).

### Protein expression and purification

*E. coli* BL21(DE3)∆SlyD strain containing the pRARE2 plasmid and expressing GST-tagged RPGRIP1L constructs were cultivated in TB medium containing 100 μg/mL ampicillin and 34 μg/mL chloramphenicol. Protein expression was induced at OD_600_ ~0.6 with 0.1 mM IPTG during 16 h at 18 °C, 200 rpm. Cells were harvested and resuspended in lysis buffer (50 mM HEPES, 150 mM NaCl, 5% *(v/v)* glycerol and pH 7.2) containing 0.1 mg/mL lysozyme, free-EDTA SigmaFast Protease Inhibitor cocktail (Sigma-Aldrich) and 50 μg/mL DNAse (Sigma-Aldrich).

Cells were disrupted by sonication and centrifuged at 40,000 x g. The supernatant was loaded onto a 5 mL GSTrap FF column (GE Healthcare), pre-equilibrated with lysis buffer, using an Äkta FPLC (GE Healthcare). GST-tagged constructs were eluted using lysis buffer added by 10 mM reduced glutathione (Sigma-Aldrich). The recombinant protein was dialyzed against ligation buffer (50 mM HEPES, 20 mM NaCl, 5% *(v/v)* glycerol, pH 7.5) and subsequently incubated with 1% *(m/m)* trypsin (Sigma-Aldrich), at 4 °C, during 30 min under gentle agitation, for GST-tag cleavage. The reaction was stopped with 1 mM phenylmethylsulfonyl fluoride (PMSF) (USB Corporation, Cleveland, OH) and loaded onto a 5 mL HiTrap Q FF column (GE Healthcare) pre-equilibrated with ligation buffer using an Äkta FPLC (GE Healthcare). After washing the resin, the target protein was eluted using a step-gradient from 20 mM to 1000 mM NaCl. Residual contamination with GST was removed by affinity chromatography using a 5 mL GSTrap FF column (GE Healthcare). All purification steps were carried out at 4 °C. MyoVa-GTD was expressed and purified as described by Nascimento and co-workers^30^. All proteins were quantified by the Edelhoch method^50^ using a NanoDrop^TM^ 2000/2000c spectrophotometer (Thermo Scientific, Wilmington, DE) and analyzed by dynamic light scattering (DLS), using a ZetaSizer Nano ZS90 equipment (Malvern Instruments, Malvern, United Kingdom), in order to check the structural homogeneity.

### Microscale thermophoresis (MST)

MyoVa-GTD was incubated with three times molar excess of fluorescein isothiocyanate (FITC) (Molecular Probes, Eugene, OR) dye in buffer 50 mM HEPES, 150 mM NaCl, 5% *(v/v)* glycerol and pH 8.0 at 4 °C during 16 h under gentle agitation. The FITC-labeled MyoVa-GTD was purified and FITC excess removed using a 5 mL HiTrap Desalting column (GE Healthcare) pre-equilibrated with buffer 50 mM HEPES, 150 mM NaCl, 5% *(v/v)* glycerol and pH 7.2. Labeling efficiency was evaluated by measuring the absorbance ratio 280/495 nm using a NanoDrop^TM^ 2000/2000c spectrophotometer (Thermo Scientific).

MST assays^51^ were performed using 300 nM of FITC-labeled MyoVa-GTD and a serial dilution of RPGRIP1L-C2 domains in interaction buffer (50 mM HEPES, 150 mM NaCl, 5% *(v/v)* glycerol, 0.05% *(v/v)* tween-20 and pH 7.2). Samples were loaded into Monolith^TM^ NT.115 MST Premium Coated capillaries (NanoTemper Technologies, Munich, Germany) and thermophoresis data were measured in the Monolith^TM^ NT.115 device (NanoTemper Technologies) using a LED power of 20% and a MST power of 60%. Initial fluorescence and back diffusion were measured for 5 s whereas the thermophoretic movement was recorded for 30 s. All assays were performed in triplicate and data were processed using the NTAffinity Analysis software (NanoTemper Technologies) and Origin 8.0. The dissociation constant (*K*_d_) was calculated from changes in the normalized fluorescence (F_norm_) as a function of the RPGRIP1L-C2 domains concentration.

### *In situ* Proximity Ligation Assay (PLA)

Human hTERT RPE-1 cells (ATCC, Manassas, VA) were cultivated at 37 °C and 5% *(v/v)* CO_2_ in Dulbecco Modified Eagle medium (DMEM) supplied with HAM F-12 nutrient (Sigma-Aldrich) and 10% *(v/v)* fetal bovine serum (Life Technologies, Carlsbad, CA). Cells at concentration 1,500 cells/mL were cultivated on 13 mm diameter cover slips (Knittel, Braunschweig, Germany) into 24-well plates (Corning incorporated Costar^®^, Corning, NY) for 24 h. To induce primary cilium formation, confluent cells were cultivated for additional 24 h in medium without fetal bovine serum. For the confocal analysis, cells were fixed with 3.7% *(v/v)* formaldehyde solution (Sigma-Aldrich) in PBS pH 7.4 for 10 min, washed with 1% *(w/v)* BSA (Sigma-Aldrich), 0.2% *(v/v)* Triton X-100 (Sigma-Aldrich) in PBS pH 7.4 (wash buffer) and permeabilized with 0.5% *(v/v)* Triton X-100 in PBS pH 7.4 for 10 min. Free aldehydes were blocked by incubating cells with 10 mM glycine (Promega, Madson, WI) in PBS pH 7.4 for 5 min. Cells were blocked with 3% *(w/v)* BSA, 0.2% *(v/v)* Triton X-100 in PBS pH 7.4 for 30 min. PLA was performed using rabbit anti-MyoVa at 0.2 μg/mL (M4812, Sigma-Aldrich), goat anti-RPGRIP1L at 1 μg/mL (sc-165400, Santa Cruz) and Duolink^®^ *in situ* red starter kit goat/rabbit (DUO92105, Sigma-Aldrich) whereas counterstaining used DAPI, mouse anti-acetylated-α-tubulin at 0.7 μg/mL (32–2700, Invitrogen, Thermo Fisher Scientific) and chicken anti-mouse 488 at 10 μg/mL (A21200, Invitrogen, Thermo Fisher Scientific) antibodies. All procedures were performed according to the manufacturer’s protocol. ProLong^®^ Gold Antifade (Life Technologies) was used as mounting medium. Cells were imaged in True Confocal Scanning (TCS) SP8 microscope (Leica, Wetzlar, Germany) at the Biological Imaging facility from the Brazilian Biosciences National Laboratory (LNBio). Images were obtained using oil immersion HC PL APO CS2 63x/1.4 objective lens and 1.4 numerical aperture. Microscopy images were previously analyzed in the LAS AF lite program (Leica). The maximum intensity projection and channel levels correction were performed using the FIJI platform^52^.

### Dominant-negative overexpression

For dominant-negative overexpression assays, 3 × 10^4^ hTERT RPE-1 cells (ATCC) were incubated overnight on 13 mm diameter cover slips (Knittel, Braunschweig, Germany) into 24-well plates (Corning incorporated Costar^®^). The cells were transfected using Lipofectamine^®^ 3000 (Thermo Fisher Scientific, Carlsbad, CA). For overexpression, pEGFP-C1 plasmids encoding chicken brain MyoVa-mGTD (residues 1377-1830; CAA77782.1) or MyoVa-GTD (residues 1423–1830; CAA77782.1) fused to EGFP as well as the empty plasmid encoding only EGFP as control were used. MyoVa-mGTD was identical to the one described in^19^,^20^, except that the insert, previously in pS65T-C1, was transferred to pEGFP-C1. MyoVa-GTD (residues 1423–1830; CAA77782.1) was PCR amplified using as template a chicken brain MyoVa full tail cDNA clone^53^, and the PCR product was inserted into pEGFP-C1 plasmid in fusion with EGFP. The transfections were carried out in a final volume of 500 μL medium following the manufactures’ instructions. After 72 h of incubation for overexpression of EGFP or EGFP-MyoVa-GTD, cells were washed with PBS, fixed with 4% *(v/v)* paraformaldehyde pH 7.4 for 15 min, washed with PBS and processed for immunofluorescence and data acquisition as described above. For these assays, cells were imaged using a Leica CTR 6000 microscope (Leica).

## Additional Information

**How to cite this article**: Assis, L. H. P. *et al*. The molecular motor Myosin Va interacts with the cilia-centrosomal protein RPGRIP1L. *Sci. Rep.* **7**, 43692; doi: 10.1038/srep43692 (2017).

**Publisher's note:** Springer Nature remains neutral with regard to jurisdictional claims in published maps and institutional affiliations.

## Supplementary Material

Supplementary Information

## References

[b1] Trybus K. M. Myosin V from head to tail. Cellular and molecular life sciences: CMLS 65, 1378–1389, doi: 10.1007/s00018-008-7507-6 (2008). 18239852 PMC2613318

[b2] Odronitz F. & Kollmar M. Drawing the tree of eukaryotic life based on the analysis of 2,269 manually annotated myosins from 328 species. Genome biology 8, R196, doi: 10.1186/gb-2007-8-9-r196 (2007). 17877792 PMC2375034

[b3] Hammer J. A. 3rd & Sellers J. R. Walking to work: roles for class V myosins as cargo transporters. Nature reviews. Molecular cell biology 13, 13–26, doi: 10.1038/nrm3248 (2012). 22146746

[b4] Hammer J. A. 3rd & Wagner W. Functions of class V myosins in neurons. J Biol Chem 288, 28428–28434, doi: 10.1074/jbc.R113.514497 (2013). 23990471 PMC3789944

[b5] Berg J. S., Powell B. C. & Cheney R. E. A millennial myosin census. Molecular biology of the cell 12, 780–794 (2001). 11294886 10.1091/mbc.12.4.780 PMC32266

[b6] Rudolf R., Bittins C. M. & Gerdes H. H. The role of myosin V in exocytosis and synaptic plasticity. Journal of neurochemistry 116, 177–191, doi: 10.1111/j.1471-4159.2010.07110.x (2011). 21077886

[b7] Pastural E. . Griscelli disease maps to chromosome 15q21 and is associated with mutations in the myosin-Va gene. Nature genetics 16, 289–292, doi: 10.1038/ng0797-289 (1997). 9207796

[b8] Evans R. D. . Myosin-Va and dynamic actin oppose microtubules to drive long-range organelle transport. Current biology: CB 24, 1743–1750, doi: 10.1016/j.cub.2014.06.019 (2014). 25065759 PMC4131108

[b9] Bittins C. M., Eichler T. W. & Gerdes H. H. Expression of the dominant-negative tail of myosin Va enhances exocytosis of large dense core vesicles in neurons. Cellular and molecular neurobiology 29, 597–608, doi: 10.1007/s10571-009-9352-z (2009). 19214741 PMC11505827

[b10] Bittins C. M., Eichler T. W., Hammer J. A. 3rd & Gerdes H. H. Dominant-negative myosin Va impairs retrograde but not anterograde axonal transport of large dense core vesicles. Cellular and molecular neurobiology 30, 369–379, doi: 10.1007/s10571-009-9459-2 (2010). 19787448 PMC3878150

[b11] Wagner W., Brenowitz S. D. & Hammer J. A. 3rd. Myosin-Va transports the endoplasmic reticulum into the dendritic spines of Purkinje neurons. Nature cell biology 13, 40–48, doi: 10.1038/ncb2132 (2011). 21151132 PMC3403743

[b12] van Diepen M. T. . MyosinV controls PTEN function and neuronal cell size. Nature cell biology 11, 1191–1196, doi: 10.1038/ncb1961 (2009). 19767745 PMC2756284

[b13] Lise M. F. . Myosin-Va-interacting protein, RILPL2, controls cell shape and neuronal morphogenesis via Rac signaling. Journal of cell science 122, 3810–3821, doi: 10.1242/jcs.050344 (2009). 19812310 PMC2758809

[b14] Shnitsar I. . PTEN regulates cilia through Dishevelled. Nature communications 6, 8388, doi: 10.1038/ncomms9388 (2015). PMC4598566 26399523

[b15] Schaub J. R. & Stearns T. The Rilp-like proteins Rilpl1 and Rilpl2 regulate ciliary membrane content. Molecular biology of the cell 24, 453–464, doi: 10.1091/mbc.E12-08-0598 (2013). 23264467 PMC3571868

[b16] Fliegauf M., Benzing T. & Omran H. When cilia go bad: cilia defects and ciliopathies. Nature reviews. Molecular cell biology 8, 880–893, doi: 10.1038/nrm2278 (2007). 17955020

[b17] Lee J. H. & Gleeson J. G. The role of primary cilia in neuronal function. Neurobiology of disease 38, 167–172, doi: 10.1016/j.nbd.2009.12.022 (2010). 20097287 PMC2953617

[b18] Knodler A. . Coordination of Rab8 and Rab11 in primary ciliogenesis. Proceedings of the National Academy of Sciences of the United States of America 107, 6346–6351, doi: 10.1073/pnas.1002401107 (2010). 20308558 PMC2851980

[b19] Espreafico E. M. . Localization of myosin-V in the centrosome. Proceedings of the National Academy of Sciences of the United States of America 95, 8636–8641 (1998). 9671730 10.1073/pnas.95.15.8636 PMC21128

[b20] Tsakraklides V. . Subcellular localization of GFP-myosin-V in live mouse melanocytes. Journal of cell science 112 (Pt 17), 2853–2865 (1999). 10444380 10.1242/jcs.112.17.2853

[b21] Lionne C., Buss F., Hodge T., Ihrke G. & Kendrick-Jones J. Localization of myosin Va is dependent on the cytoskeletal organization in the cell. Biochemistry and cell biology=Biochimie et biologie cellulaire 79, 93–106 (2001). 11235920

[b22] Wu X., Kocher B., Wei Q. & Hammer J. A. 3rd. Myosin Va associates with microtubule-rich domains in both interphase and dividing cells. Cell motility and the cytoskeleton 40, 286–303, doi: 10.1002/(SICI)1097-0169(1998)40:3<286::AID-CM7>3.0.CO;2-B (1998). 9678671

[b23] Doxsey S. Re-evaluating centrosome function. Nature reviews. Molecular cell biology 2, 688–698, doi: 10.1038/35089575 (2001). 11533726

[b24] Farina F. . The centrosome is an actin-organizing centre. Nature cell biology 18, 65–75, doi: 10.1038/ncb3285 (2016). 26655833 PMC4880044

[b25] Delous M. . The ciliary gene RPGRIP1L is mutated in cerebello-oculo-renal syndrome (Joubert syndrome type B) and Meckel syndrome. Nature genetics 39, 875–881, doi: 10.1038/ng2039 (2007). 17558409

[b26] Vierkotten J., Dildrop R., Peters T., Wang B. & Ruther U. Ftm is a novel basal body protein of cilia involved in Shh signalling. Development 134, 2569–2577, doi: 10.1242/dev.003715 (2007). 17553904

[b27] Mahuzier A. . Dishevelled stabilization by the ciliopathy protein Rpgrip1l is essential for planar cell polarity. The Journal of cell biology 198, 927–940, doi: 10.1083/jcb.201111009 (2012). 22927466 PMC3432770

[b28] Arts H. H. . Mutations in the gene encoding the basal body protein RPGRIP1L, a nephrocystin-4 interactor, cause Joubert syndrome. Nature genetics 39, 882–888, doi: 10.1038/ng2069 (2007). 17558407

[b29] Remans K., Burger M., Vetter I. R. & Wittinghofer A. C2 domains as protein-protein interaction modules in the ciliary transition zone. Cell reports 8, 1–9, doi: 10.1016/j.celrep.2014.05.049 (2014). 24981858

[b30] Nascimento A. F. . Structural insights into functional overlapping and differentiation among myosin V motors. The Journal of biological chemistry 288, 34131–34145, doi: 10.1074/jbc.M113.507202 (2013). 24097982 PMC3837155

[b31] Eves P. T., Jin Y., Brunner M. & Weisman L. S. Overlap of cargo binding sites on myosin V coordinates the inheritance of diverse cargoes. The Journal of cell biology 198, 69–85, doi: 10.1083/jcb.201201024 (2012). 22753895 PMC3392941

[b32] Li X. D. . The globular tail domain puts on the brake to stop the ATPase cycle of myosin Va. Proceedings of the National Academy of Sciences of the United States of America 105, 1140–1145, doi: 10.1073/pnas.0709741105 (2008). 18216256 PMC2234105

[b33] Pylypenko O. . Structural basis of myosin V Rab GTPase-dependent cargo recognition. Proceedings of the National Academy of Sciences of the United States of America 110, 20443–20448, doi: 10.1073/pnas.1314329110 (2013). 24248336 PMC3870677

[b34] Costa M. C., Mani F., Santoro W. Jr., Espreafico E. M. & Larson R. E. Brain myosin-V, a calmodulin-carrying myosin, binds to calmodulin-dependent protein kinase II and activates its kinase activity. The Journal of biological chemistry 274, 15811–15819 (1999). 10336484 10.1074/jbc.274.22.15811

[b35] Pranchevicius M. C. . Myosin Va phosphorylated on Ser1650 is found in nuclear speckles and redistributes to nucleoli upon inhibition of transcription. Cell motility and the cytoskeleton 65, 441–456, doi: 10.1002/cm.20269 (2008). 18330901

[b36] Karcher R. L. . Cell cycle regulation of myosin-V by calcium/calmodulin-dependent protein kinase II. Science 293, 1317–1320, doi: 10.1126/science.1061086 (2001). 11509731

[b37] Sang L. . Mapping the NPHP-JBTS-MKS protein network reveals ciliopathy disease genes and pathways. Cell 145, 513–528, doi: 10.1016/j.cell.2011.04.019 (2011). 21565611 PMC3383065

[b38] Jensen V. L. . Formation of the transition zone by Mks5/Rpgrip1L establishes a ciliary zone of exclusion (CIZE) that compartmentalises ciliary signalling proteins and controls PIP2 ciliary abundance. The EMBO journal 34, 2537–2556, doi: 10.15252/embj.201488044 (2015). 26392567 PMC4609185

[b39] Noor A. . CC2D2A, encoding a coiled-coil and C2 domain protein, causes autosomal-recessive mental retardation with retinitis pigmentosa. American journal of human genetics 82, 1011–1018, doi: 10.1016/j.ajhg.2008.01.021 (2008). 18387594 PMC2427291

[b40] Brozzi F. . Molecular mechanism of myosin Va recruitment to dense core secretory granules. Traffic 13, 54–69, doi: 10.1111/j.1600-0854.2011.01301.x (2012). 21985333

[b41] Hehnly H., Chen C. T., Powers C. M., Liu H. L. & Doxsey S. The centrosome regulates the Rab11- dependent recycling endosome pathway at appendages of the mother centriole. Current biology: CB 22, 1944–1950, doi: 10.1016/j.cub.2012.08.022 (2012). 22981775 PMC3917512

[b42] Pastural E. . Two genes are responsible for Griscelli syndrome at the same 15q21 locus. Genomics 63, 299–306, doi: 10.1006/geno.1999.6081 (2000). 10704277

[b43] Juric-Sekhar G., Adkins J., Doherty D. & Hevner R. F. Joubert syndrome: brain and spinal cord malformations in genotyped cases and implications for neurodevelopmental functions of primary cilia. Acta neuropathologica 123, 695–709, doi: 10.1007/s00401-012-0951-2 (2012). 22331178

[b44] Khanna H. . A common allele in RPGRIP1L is a modifier of retinal degeneration in ciliopathies. Nature genetics 41, 739–745, doi: 10.1038/ng.366 (2009). 19430481 PMC2783476

[b45] Yoder B. Ciliary Function in Mammalian Development. (Elsevier Science, 2011).

[b46] Stratigopoulos G. . Hypomorphism for RPGRIP1L, a ciliary gene vicinal to the FTO locus, causes increased adiposity in mice. Cell metabolism 19, 767–779, doi: 10.1016/j.cmet.2014.04.009 (2014). 24807221 PMC4131684

[b47] Alborghetti M. R., Furlan A. S. & Kobarg J. FEZ2 has acquired additional protein interaction partners relative to FEZ1: functional and evolutionary implications. PloS one 6, e17426, doi: 10.1371/journal.pone.0017426 (2011). 21408165 PMC3050892

[b48] Altschul S. F., Gish W., Miller W., Myers E. W. & Lipman D. J. Basic local alignment search tool. Journal of molecular biology 215, 403–410, doi: 10.1016/S0022-2836(05)80360-2 (1990). 2231712

[b49] Lemos T. A., Passos D. O., Nery F. C. & Kobarg J. Characterization of a new family of proteins that interact with the C-terminal region of the chromatin-remodeling factor CHD-3. FEBS letters 533, 14–20 (2003). 12505151 10.1016/s0014-5793(02)03737-7

[b50] Edelhoch H. Spectroscopic determination of tryptophan and tyrosine in proteins. Biochemistry 6, 1948–1954 (1967). 6049437 10.1021/bi00859a010

[b51] Seidel S. A. . Microscale thermophoresis quantifies biomolecular interactions under previously challenging conditions. Methods 59, 301–315, doi: 10.1016/j.ymeth.2012.12.005 (2013). 23270813 PMC3644557

[b52] Schindelin J. . Fiji: an open-source platform for biological-image analysis. Nature methods 9, 676–682, doi: 10.1038/nmeth.2019 (2012). 22743772 PMC3855844

[b53] Espreafico E. M. . Primary structure and cellular localization of chicken brain myosin-V (p190), an unconventional myosin with calmodulin light chains. The Journal of cell biology 119, 1541–1557 (1992). 1469047 10.1083/jcb.119.6.1541 PMC2289763

[b54] Soding J., Biegert A. & Lupas A. N. The HHpred interactive server for protein homology detection and structure prediction. Nucleic acids research 33, W244–248, doi: 10.1093/nar/gki408 (2005). 15980461 PMC1160169

[b55] Krissinel E. & Henrick K. Secondary-structure matching (SSM), a new tool for fast protein structure alignment in three dimensions. Acta crystallographica. Section D, Biological crystallography 60, 2256–2268, doi: 10.1107/S0907444904026460 (2004). 15572779

