# Supplementary material for: The molecular motor Myosin Va interacts with the cilia-centrosomal protein RPGRIP1L
Source: Sci Rep. 2017 Mar 7;7:43692. doi: 10.1038/srep43692 (PMC5339802; doi:10.1038/srep43692)
Supplement: Supplementary Information [file srep43692-s1.pdf]

Supplementary information

The molecular motor Myosin Va interacts with the cilia-centrosomal protein RPGRIP1L

Assis, L.H.P.[1,2#], Silva-Junior, R.M.P.[3#], Dolce, L.G.[1,2], Alborghetti, M.R.[1], Honorato, R.V.[1], Nascimento, A.F.Z.[1,2], Melo-Hanchuk, T.D.[4], Trindade, D.M.[1], Tonoli, C.C.C.[1], Santos, C.T.[3], Oliveira, P.S.L.[1], Larson, R.E.[3], Kobarg, J.[4], Espreafico, E.M.[3], Giuseppe, P.O.[1*], Murakami, M.T.[1*]

Table S1. Human MyoVa-interacting proteins identified by the yeast two-hybrid screen.

Gene	NCBI accession number	Protein description[1]	Coded protein residues retrieved (complete)[2]	Incidence in the screening
AES	NP_945320.1	Amino-terminal enhancer of split	1-30 (264)	1
CC2D1A	NP_060191.3	Coiled-coil and C2 domain containing	502-663 (951)	1
CCAR1	NP_060707.2	Cell division cycle and apoptosis regulator 1	247-399 (1150)	1
FTH1	NP_002023.2	Ferritin, heavy polypeptide 1	1-134 (183)	15
IMP3	NP_060755.1	U3 small nucleolar ribonucleoprotein	67-184 (184)	4
NPNT	EAX06195.1	Nephronectin	243-260 (319)	1
PPP1R1A	NP_006732.3	Protein phosphatase 1, regulatory subunit 1A	1-135 (171)	1
RPGRIP1L	NP_001295263.1	RPGRIP1-like	1-119 (1269)	1
RPS2P31	AAH04520.2	Similar to 40S ribosomal protein S2	1-97 (97)	1
RPS7	NP_001002.1	40S Ribosomal protein S7	1-173 (194)	3
WASH1	NP_878908.4	WAS protein family homolog 1	55-128 (465)	1
WDR6	NP_060501.3	WD repeat domain	1.072-1151 (1151)	3
YY1AP1	NP_001185832.1	YY1 associated protein 1	562-711 (888)	8
ZNF428	NP_872304.2	Zinc finger protein 428	1-118 (188)	1
ZNF576	NP_077303.1	Zinc finger protein 576	1-170 (170)	1
ZNF714	NP_872321.2	Zinc finger protein 714	507-548 (555)	2

[1] Results obtained from analysis with BLASTX tool from NCBI [1].

[2] It is depicted the minimum length of the retrieved sequences which could be visualized by foward DNA sequencing only.

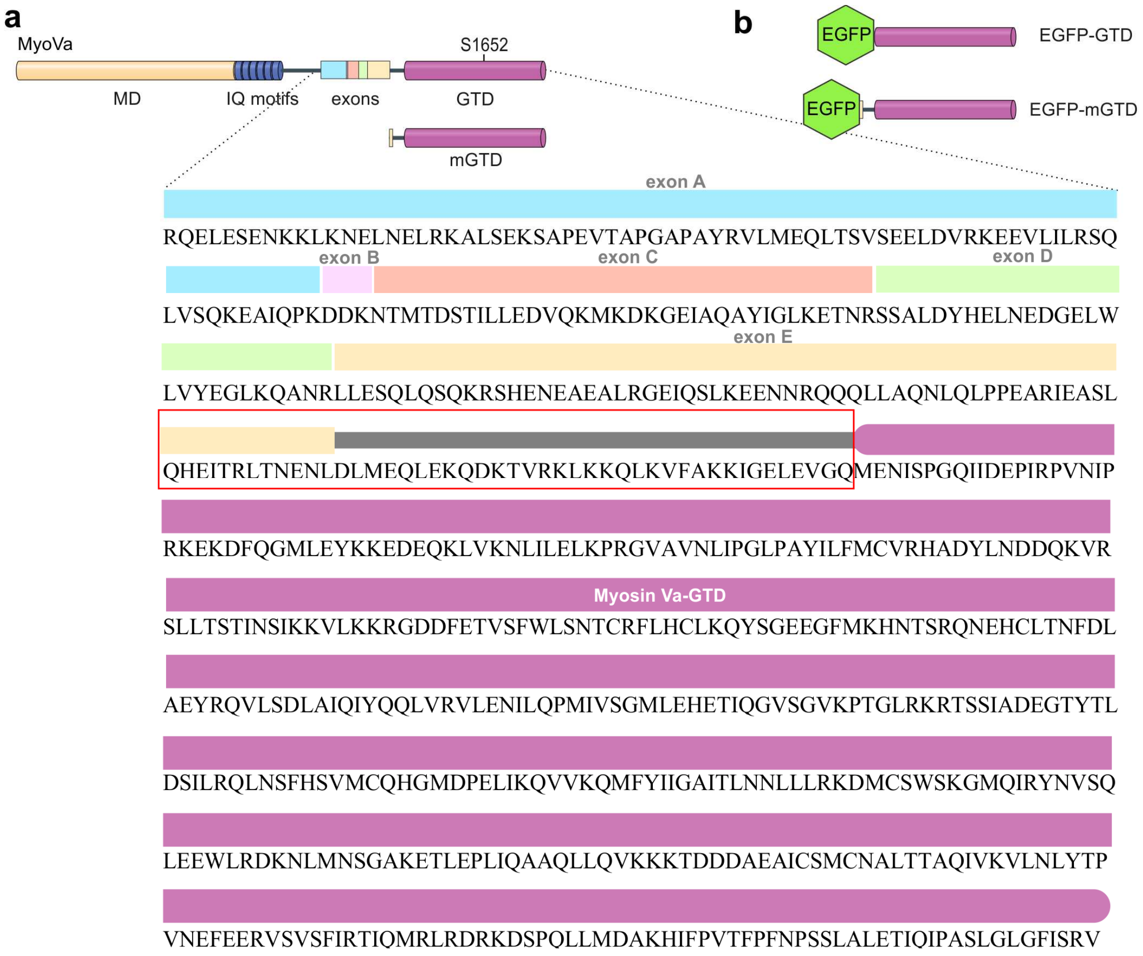

Figure S1. Myosin Va constructs used in dominant-negative studies. (a) Schematic representation of MyoVa structure highlighting the tail region comprising the exons that can be alternatively spliced (A-E, exon F was omitted), a linker region (gray) and the GTD (magenta) whose crystal structure has been determined (PDB: 4J5L). (b) For dominant negative studies, we used pEGFP-C1 plasmids encoding chicken brain MyoVa-GTD$^{aa1423\text{-}1830}$ (MEN-SRV) or MyoVa-mGTD$^{aa1377\text{-}1830}$ (QHE-SRV) fused with EGFP. MyoVa-mGTD includes 45 amino acids (red box) upstream the GTD and was identical to the one already described[2,3], except that the insert, previously in pS65T-C1, was transferred to pEGFP-C1.

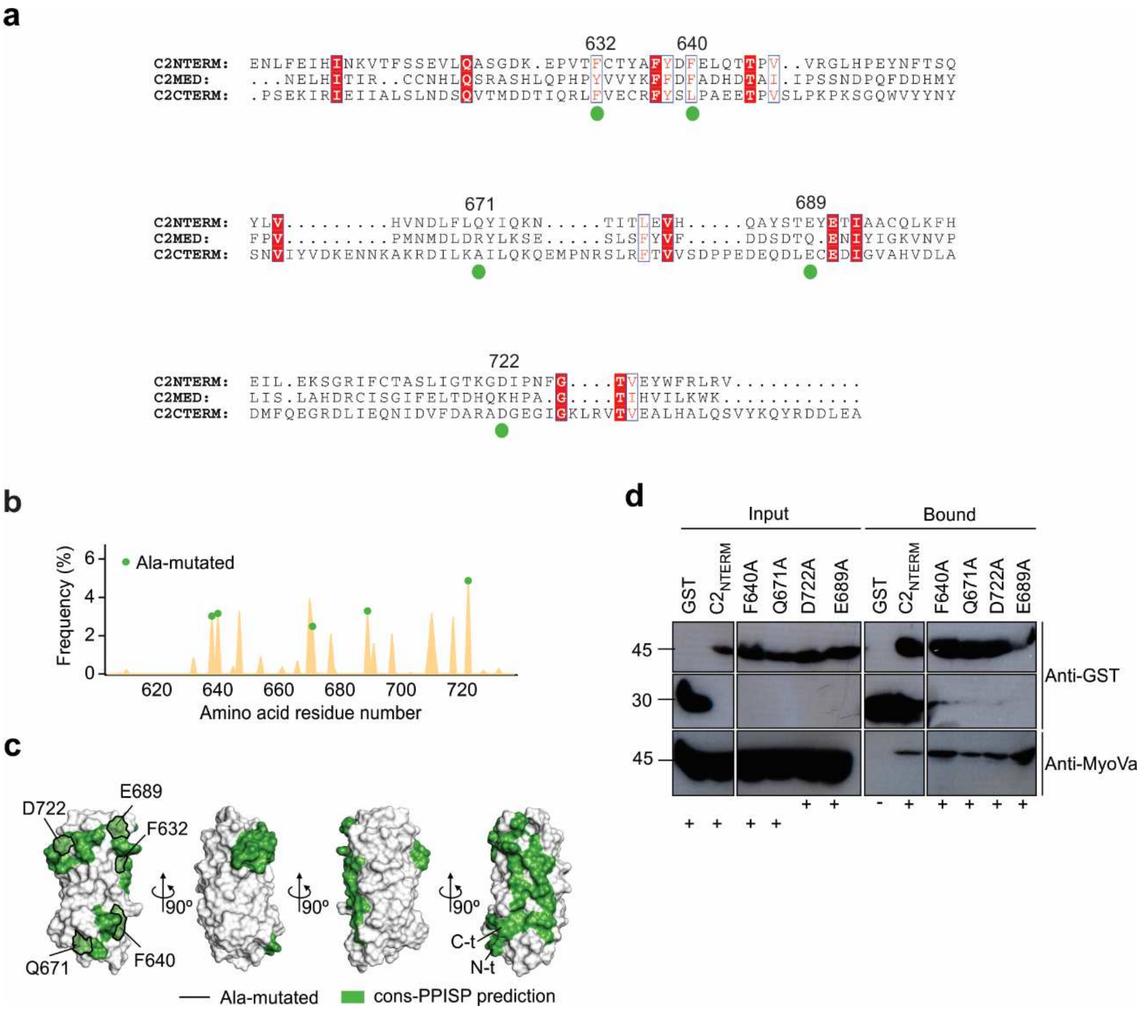

Figure S2. Attempts to identify the molecular basis for MyoVa-recognition by the C2 domains of RPGRIP1L. *(a)* Sequence comparison between the three C2 domains of RPGRIP1L highlighting identical (red boxes) and similar (red letters) residues as well as those predicted to interact with MyoVa-GTD according to *in silico* studies (panels b and c). Note that most of the predicted residues selected for site-directed mutagenesis (632, 640, 689, 722) seem to be semi-conserved. (*b*) The frequency in which the $C2_{NTERM}$ residues appeared in contact with MyoVa-GTD surface in molecular docking analysis. Green dots represent selected residues for mutation studies aiming to find key residues for MyoVa-GTD recognition. *(c)* $C2_{NTERM}$ surface (PDB ID: 2YRB) highlighting potential protein-protein interaction sites (green) identified by cons-PPISP analysis [4]. The residues F632, F640, Q671, E689 and D722 were selected to be Ala-mutated because they meet both criteria of potential protein-protein interaction sites and high frequency of contact to MyoVa-GTD in molecular docking analysis (panel b). Further details about these *in silico* analysis are described below. (*d*) In pull-down assays, four Ala-mutants do not abolish the interaction between MyoVa-GTD and RPGRIP1L-$C2_{NTERM}$. The F632A

mutant was unstable in solution, precluding the evaluation of its effect in MyoVa-GTD binding.

Supplementary methods

In silico molecular docking

Molecular docking was performed using the atomic coordinates of MyoVa-GTD (PDB ID: 4J5L; [5]) and RPGRIP1L-C2$_{NTERM}$ (PDB ID: 2YRB). The W1713, Y1721, Q1755 and F1792 residues of MyoVa were considered as restriction points, since they play a role in the interaction, according to our YTH and site-directed mutagenesis data. A two-step algorithm was used to sample possible interacting conformations between the target proteins. This algorithm was developed *in-house* and takes advantage of a software driven by geometric complementarity[6] and robust energy evaluation scores[7]. Each binding partner is characterized as a Connolly dot surface; restricted input residues numbers are related with atom numbers and formatted accordingly. Subsequent to the docking based on geometric complementarity, resulting decoys are submitted to the energetic step. The initialization of the energetic routine of the docking protocol uses a random seed, henceforth one of the most important parameters to be defined is the ratio between initializations and output decoys. For this task, default values were set at 600 initializations generating 10 decoys of local optimization. The pipeline has been optimized to distribute each initialization to an available processing core. A custom script was developed to evaluate intermolecular contact frequency based on the overall complex binding score. A total of 6,000 decoys were generated for each algorithm execution. The most frequent contacts that exist within the first quartile of the binding score distribution were identified for each decoy and described accordingly. This result was compared with potential protein-binding sites at C2$_{NTERM}$ structure, predicted using a consensus neural

network method (cons-PPISP)[4], to select, for mutagenesis studies, residues potentially involved in MyoVa-GTD binding.

Site-directed mutagenesis of $C2_{NTERM}$ domain

C_{2NTERM} mutants (F632A, F640A, Q671A, E689A and D722A) were generated using the QuikChange site-directed mutagenesis kit (Agilent Technologies, Santa Clara, CA).

Supplementary references

1 Altschul, S. F. *et al.* Gapped BLAST and PSI-BLAST: a new generation of protein database search programs. *Nucleic acids research* **25**, 3389-3402 (1997).

2 Tsakraklides, V. *et al.* Subcellular localization of GFP-myosin-V in live mouse melanocytes. *Journal of cell science* **112 (Pt 17)**, 2853-2865 (1999).

3 Espreafico, E. M. *et al.* Localization of myosin-V in the centrosome. *Proceedings of the National Academy of Sciences of the United States of America* **95**, 8636-8641 (1998).

4 Chen, H. & Zhou, H. X. Prediction of interface residues in protein-protein complexes by a consensus neural network method: test against NMR data. *Proteins* **61**, 21-35, doi:10.1002/prot.20514 (2005).

5 Nascimento, A. F. *et al.* Structural insights into functional overlapping and differentiation among myosin V motors. *The Journal of biological chemistry* **288**, 34131-34145, doi:10.1074/jbc.M113.507202 (2013).

6 Duhovny, D., Nussinov, R. & Wolfson, H. J. in *Algorithms in Bioinformatics: Second International Workshop, WABI 2002 Rome, Italy, September 17–21, 2002 Proceedings* (eds Roderic Guigó & Dan Gusfield) 185-200 (Springer Berlin Heidelberg, 2002).

7 Gray, J. J. *et al.* Protein-protein docking with simultaneous optimization of rigid-body displacement and side-chain conformations. *Journal of molecular biology* **331**, 281-299 (2003).
